# The Healthy Diabetes Plate

**Published:** 2006-12-15

**Authors:** Martha Raidl, Kristina Spain, Mimi Hartman-Cunningham, Rhea Lanting, Marsha Lockard, Shelly Johnson, Marnie Spencer, Laura Sant, Julia Welch, Audrey Liddil

**Affiliations:** University of Idaho; Bureau of Clinical and Preventive Services, Idaho Department of Health and Welfare, Boise, Idaho; Mimi Hartman-Cunningham, Idaho Diabetes Prevention and Control Program, Idaho Department of Health and Welfare, Boise, Idaho; Idaho Diabetes Prevention and Control Program, Idaho Department of Health and Welfare, Boise, Idaho; University of Idaho (UI), Twin Falls, Idaho; UI, Marsing, Idaho; UI, Coeur d'Alene, Idaho; UI, Blackfoot, Idaho; UI, Preston, Idaho; UI, Grangeville, Idaho. Ms. Welch is now affiliated with Schweitzer Engineering, Pullman, Wash; UI, Pocatello, Idaho

## Abstract

**Background:**

Diabetes education presents two major challenges to the U.S. Cooperative Extension System. The first is that the majority of diabetes education services are provided in more populated areas, resulting in large nonurban areas being underserved. The second is that many individuals with diabetes find the meal-planning component of diabetes education confusing.

**Context:**

The University of Idaho, a land-grant institution, includes teaching, research, and extension as part of its mission. Extension means "reaching out," and in Idaho, the Extension Service provides research-based programs on agricultural, natural resources, youth, family, community, and environmental issues in 42 of Idaho's 44 counties, making it accessible to most Idahoans.

**Methods:**

The University of Idaho Extension Service collaborated with dietitians and certified diabetes educators to develop and test materials that simplify the meal-planning component of diabetes education. The result was a four-lesson curriculum, The Healthy Diabetes Plate, which used the plate format to teach individuals about the type and amount of foods they should consume at each meal. In 2004, the four-lesson curriculum was taught in three urban and five rural counties. Surveys, hands-on activities, and note-taking of participants' comments were used to collect data on participants' characteristics, their ability to plan meals, and changes in eating habits.

**Consequences:**

Participants were able to correctly plan breakfast, lunch, and dinner meals and improved their intake of fruit and vegetables.

**Interpretation:**

Quantitative and qualitative evaluation information gathered from class participants helped identify which components of The Healthy Diabetes Plate curriculum were effective.

## Background

Approximately 21 million Americans, or 7% of the American population, have been told by a doctor or other health care professional that they have diabetes (1). But how well do people with diabetes manage their disease? A survey conducted by the American Association of Clinical Endocrinologists on 157,000 individuals with type 2 diabetes found that approximately two thirds did not have their diabetes under control and were more likely to experience blindness and limb loss or die prematurely from myocardial infarction, kidney failure, or a stroke (2).

Diabetes education is recommended to help individuals control their diabetes, but to be effective diabetes education must be accessible and understandable. Currently, most diabetes educators are located in health care organizations in urban areas (3), and most diabetes information provided in nutrition therapy classes is poorly understood by participants (4). In Idaho, the solution to both problems was to have the University of Idaho (UI) Extension Service deliver diabetes education. The extension service is accessible to rural and urban clientele, having offices in 42 of 44 counties in Idaho. To make nutrition therapy classes understandable to participants, materials were developed using a visual format, the Idaho Plate Method (IPM). A group of Idaho dietitians modified the Swedish Plate Method, which has been used successfully since 1987 to teach meal planning to individuals with type 2 diabetes (5-7). The IPM follows the nutritional guidelines of the American Diabetes Association and the American Dietetic Association (8).

Studies show that participants gain and retain more knowledge about diabetes when attending more than one lesson (9). Although the IPM has been used as a tool to plan meals, no multi-lesson diabetes curriculum had been developed for using the IPM as part of the meal-planning process. Therefore, a curriculum was developed that focused on using the IPM to plan meals in many settings. The resulting four-lesson curriculum was called The Healthy Diabetes Plate (10), and its target audience was defined as adults with type 2 diabetes or their caregivers. The purpose of this project was to develop, test, and evaluate The Healthy Diabetes Plate curriculum that could be taught by extension educators to underserved populations.

## Context

The incidence of diabetes in Idaho has increased from 4% in 1997 to 6.2% in 2004 (11). In 2005, more than 61% of the state's area or population was designated as medically underserved (12). The UI Extension System can help reach this underserved population. UI faculty members who teach extension programs in the community are called extension educators, and those that teach nutrition and health programs are called Family and Consumer Sciences (FCS) extension educators. The extension nutrition education specialist at UI provides research-based information and program development for FCS extension educators.

The increased incidence in diabetes in Idaho has created greater interest and demand for diabetes education materials among FCS extension educators. In 2000, the UI extension nutrition education specialist conducted a needs assessment with 24 UI FCS extension educators. The number one request among the extension educators was for new materials on nutrition for diabetes education. The request was based on input from advisory boards and residents in their local communities. The UI FCS extension educators requested that any diabetes education materials developed include basic information on diabetes but focus mainly on meal planning in a variety of settings. The UI College of Agricultural and Life Sciences provided a $3000 grant to develop and test diabetes materials that could be used by UI FCS extension faculty.

It was also important that materials used by extension faculty be technically accurate. The program manager for the Idaho Diabetes Prevention and Control Program promoted The Healthy Diabetes Plate curriculum in Idaho and other states by reviewing materials and facilitating collaboration between local registered dietitians, certified diabetes educators, and FCS extension educators.

The resulting four-lesson curriculum was developed and piloted during 3 years in three states (Idaho, Oregon, and Colorado) and reviewed by 10 extension educators, three extension nutrition specialists, and three certified diabetes educators. Numerous revisions were made on the content, activities, and evaluation tools. The final peer-reviewed curriculum, called The Healthy Diabetes Plate, was published in January 2003 (10). Eight UI extension faculty members received a copy of the final curriculum in October 2003 and were trained on the materials, activities, evaluation tools, and research protocol. Testing of The Healthy Diabetes Plate was conducted during 2003 and 2004.

## Methods

### Program and activities

**Figure 1:**
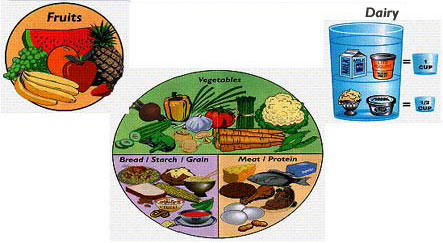
The Idaho Plate Method for meal planning, reproduced by permission from Idaho Plate Method, LLC (14).

The Healthy Diabetes Plate curriculum contains four lessons; participants met weekly either in a classroom or supermarket. Each lesson focused on teaching participants how to plan meals correctly using the IPM (Figure 1). The program was designed to reach an audience of people with diabetes and individuals who are caregivers of people with diabetes. Because individuals aged 45 years and older have an increased risk of developing diabetes, the program participants were divided into two age groups: individuals aged less than 45 years and individuals aged 45 years or older (13).

**Figure 2:**
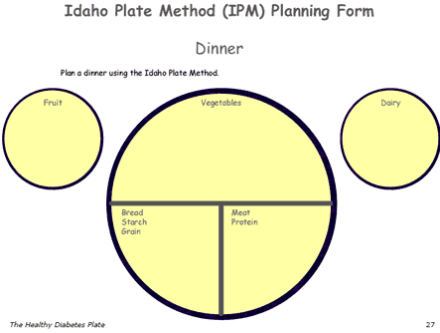
Example of a meal-planning form from The Healthy Diabetes Plate curriculum, Idaho Plate Method.

Lesson 1 covered basic information on diabetes (signs and symptoms) and an introduction to the IPM. Information about the five food groups (vegetables, starches, meats and other proteins, fruits, and dairy) and how they fit on the plate was discussed. In this class, participants were divided into three groups and instructed to plan a breakfast, lunch, or dinner meal using a meal-planning sheet (Figure 2). In lessons 2, 3, and 4, participants learned how to plan meals in three different settings. The first setting was in the home, using foods they typically ate at home; the second setting was in the supermarket, using new foods introduced during the supermarket tour; the third setting was in a restaurant or fast-food establishment. The meal-planning lessons not only reinforced the five food groups but also helped participants visually plan meals; participants were able to visualize the types and amounts of foods allowed and how the foods formed a balanced and nutritious meal.

### Recruitment of participants

Adult participants were recruited through the county extension newsletter and selected through nonrandom sampling. Extension faculty found that registration for the classes filled up quickly, and many potential participants were placed on a waiting list for the next set of classes. Participants were recruited from five rural and three urban counties. When participants residing in an urban area called to register for the class, several commented that either they could not afford the cost of diabetes education classes at their own local hospital or that they had taken a class but did not understand most of it. Residents in rural counties commented that this was the first time diabetes classes had been offered in their county. The UI Human Assurances Committee approved this study, and each participant signed a subject consent form.

### Program evaluation

The Healthy Diabetes Plate program was evaluated using pre- and postcurriculum surveys and meal-planning activities. A precurriculum survey was filled out during lesson 1 and contained questions on demographics and diabetes history. The precurriculum survey also included a four-question semiquantitative food frequency survey. Questions in the food frequency survey were related to foods promoted in the IPM — whole grains, fruits, vegetables, and milk — that are known to affect blood glucose levels (15-17). The postcurriculum survey was completed at the end of lesson 4; it included the same four-question semiquantitative food frequency survey as well as a comments section. In lessons 2, 3, and 4, a meal-planning activity was used to determine participants' abilities to plan meals correctly using the IPM. Each participant planned breakfast, lunch, and dinner meals in three different settings (i.e., home, supermarket, and eating out). The breakfast, lunch, and dinner meals were combined within each setting and analyzed. For each setting, the percentage of correct responses for each of the five food groups was calculated.

### Statistical analysis

Data were gathered on participant demographic characteristics, the reasons given by participants for taking the class, food consumption frequency, and the ability of participants to plan meals correctly in three settings. Pre- and postcurriculum food frequency questions were analyzed using *t* tests; participants served as their own control.

## Consequences

### Demographic and diabetes characteristics

Table 1 shows participants' age, sex, racial and ethnic characteristics, and reasons for taking the class. One hundred and thirty five individuals started the project, and 117 (87%) completed all four lessons. Most of the participants (88%) were aged 45 years or older. Ages ranged from 26 years to 83 years. Of the 117 participants who completed the lessons, 83% were female and 17% were male.

Table 1 also shows that 113 (96.6%) were white, and the rest of the participants were evenly divided into Hispanic, African American, Asian American, and American Indian groups. In Idaho, the most recent census data (2004) indicated that approximately 91% of the population of Idaho is white, followed by 8% Hispanics, 1.4% American Indian and Alaska Native, 1% Asian, and less than 0.5% African American, Native Hawaiian and Other Pacific Islander. While Hispanic participation in the program was low, the rest of the research population was similar to the most recent census data on Idaho (18).

Reasons why participants took the classes were divided into three categories: 1) 48% had been diagnosed with type 2 diabetes; 2) 46% did not have diabetes and were caregivers of individuals who had diabetes; and 3) 6% were not sure if they had diabetes but were interested in learning more about diabetes. The results indicate that the target population was reached.

### Eating habits

Table 2 shows that there were no significant changes in daily whole grain or milk consumption, but there were significant increases in daily fruit (*P* = .02) and vegetable consumption (*P* = .01).

### Ability to plan meals correctly

Table 3 shows that a high percentage of participants were able to plan their meals correctly: 86% to 97% of participants in the home setting; 88% to 96% in the supermarket setting; and 90% to 99% in the restaurant and fast-food setting. In all three settings, the dairy group had the lowest percentage of correct responses. Comments from the instructors and participants indicated that some participants forgot that with the IPM, cheese was grouped with meat and other protein and not with dairy.

## Interpretation

The results from the study indicate that having FCS extension educators teach The Healthy Diabetes Plate curriculum solves two problems encountered in diabetes education — understandability and accessibility. The IPM was easy for participants to understand; a high percentage of participants planned meals correctly in three different settings. Because extension faculty members are located in urban and rural counties, diabetes education became accessible to the underserved population. Also, participants in our study indicated that they preferred attending extension classes rather than visiting their physician or attending hospital classes. We speculate that extension educators may be viewed as less threatening than health professionals, and a greater level of comfort among participants may help to explain the low attrition rate in this study.

The IPM focused on meal planning and showed participants how to include all foods in their meal planning. A focus on meal planning provided an opportunity to reinforce basic information at each lesson. Exposing participants to a variety of settings helped them plan their meals accordingly. Planning meals in supermarkets and restaurants made the lessons more applicable and interesting and sparked discussions on how participants could eat out at their favorite restaurant and still adhere to the guidelines of the IPM.

Most Americans do not consume the recommended five to nine daily servings of fruits and vegetables. The supermarket tour lesson provided an excellent opportunity for participants to learn about new fruits and vegetables and how to incorporate them in their meal plan. The significant increase in fruit and vegetable consumption indicated that participants were making some of these changes in their diet.

The Healthy Diabetes Plate curriculum became a starting point for diabetes education for many participants. One outcome was that many participants wanted to continue to meet on a monthly or bimonthly basis as a support group to help one another follow the IPM. Participants also had questions about diabetes that were outside the expertise of the extension educator (e.g., questions about insulin, foot care, and matching insulin to food). The extension educators invited health care professionals such as pharmacists, podiatrists, and certified diabetes educators to answer questions at meetings. One topic not covered in The Healthy Diabetes Plate curriculum was physical activity. Many participants expressed an interest in being more physically active and starting a walking program.

One of the best ways to learn about the effectiveness of a lesson is to listen to comments made by participants. Participants made the following comments: "This is so easy to understand," "I'll be able to follow this forever," "Now I know how to fit all foods into my diet," and "I don't have to give up cookies forever."

Future projects include adding a physical activity component to the study and making The Healthy Diabetes Plate more culturally appropriate to the Hispanic population. Based on research indicating that diet and physical activity may help control and prevent type 2 diabetes, a walking program and resistance activity component are being piloted along with The Healthy Diabetes Plate lessons. One of the fastest-growing population groups in Idaho is the Hispanic population. A separate curriculum written for the Hispanic population needs to be developed.

By participating in this project, the UI Extension Service strengthened its ties with the residents in the community, health care professionals, and the Idaho Diabetes Prevention and Control Program. These diabetes education classes are now offered on a regular basis (3 to 4 times per year) in various counties to meet the needs of their clientele. Many extension faculty members serve on local diabetes advisory councils, and some health care professionals regularly refer their clients with diabetes to these classes. Finally, the manager of the Idaho Diabetes Prevention and Control Program is currently providing expertise and resources for a new diabetes pedometer project conducted by the UI Extension Service.

## Figures and Tables

**Table 1:** Participant Characteristics (N = 117), The Healthy Diabetes Plate Curriculum, Five Rural and Three Urban Counties in Idaho

Characteristic	No. of Participants (%)
**Age, y**	
<45 (range, 26-44)	14 (12.0)
≥45 (range, 45-83)	103 (88.0)
**Sex**	
Male	20 (17.1)
Female	97 (82.9)
**Race and ethnicity**	
White	113 (96.6)
Hispanic	1 (0.8)
African American	1 (0.8)
Asian American	1 (0.8)
American Indian	1 (0.8)
**Reason for taking class**	
Diagnosed with type 2 diabetes	56 (47.9)
Not diagnosed with diabetes, but are caregivers of individuals with diabetes	54 (46.2)
Not sure if they had diabetes, but are interested in learning about diabetes	7 (6.0)

**Table 2:** Results of Pre- and Postcurriculum Food Frequency Surveys (N = 117), The Healthy Diabetes Plate Curriculum, Five Rural and Three Urban Counties in Idaho

Food Group	Precurriculum Mean No. of Servings per Day (SD)	Postcurriculum No. of Servings per Day (SD)	*t *Test*P* Value
Fruits	1.8 (0.9)	3.2 (0.4)	.02
Milk	1.8 (1.0)	2.2 (0.8)	.20
Vegetables	1.3 (0.7)	4.1 (0.6)	.01
Whole grains	1.0 (0.4)	1.3 (0.8)	.54

**Table 3:** Percentage of Correct Responses on Food Choices, by Food Group and Meal Setting, The Healthy Diabetes Plate Curriculum, Five Rural and Three Urban Counties in Idaho

Food Group	Meal Setting		

	Home	Supermarket	Restaurant or Fast Food
Bread, starch, whole grains	92	96	99
Dairy	86	88	90
Fruits	94	90	98
Meat or other protein	97	94	99
Vegetables	93	90	99
